# A proposed model on MR elastography for predicting postoperative major complications in patients with hepatocellular carcinoma

**DOI:** 10.1259/bjro.20210019

**Published:** 2021-09-29

**Authors:** Kazu Shibutani, Masahiro Okada, Jitsuro Tsukada, Tomoko Hyodo, Kenji Ibukuro, Hayato Abe, Naoki Matsumoto, Yutaka Midorikawa, Mitsuhiko Moriyama, Tadatoshi Takayama

**Affiliations:** ^1^ Department of Radiology, Nihon University School of Medicine, Tokyo, Japan; ^2^ Department of Radiology, Kindai University school of medicine, Osaka, Japan; ^3^ Department of Digestive Surgery, Nihon University School of Medicine, Tokyo, Japan; ^4^ Department of Gastroenterology and Hepatology, Nihon University School of Medicine, Tokyo, Japan

## Abstract

**Objective:**

To develop a model for predicting post-operative major complications in patients with hepatocellular carcinoma (HCC).

**Methods:**

In all, 186 consecutive patients with pre-operative MR elastography were included. Complications were categorised using Clavien‒Dindo classification, with major complications defined as ≥Grade 3. Liver-stiffness measurement (LSM) values were measured on elastogram. The indocyanine green clearance rate of liver remnant (ICG-Krem) was based on the results of CT volumetry, intraoperative data, and ICG-K value. For an easy application to the prediction model, the continuous variables were converted to categories. Moreover, logistic regression analysis and fivefold cross-validation were performed. The prediction model’s discriminative performance was evaluated using the area under the receiver operating characteristic curve (AUC), and the calibration of the model was assessed by the Hosmer‒Lemeshow test.

**Results:**

43 of 186 patients (23.1%) had major complications. The multivariate analysis demonstrated that LSM, albumin–bilirubin (ALBI) score, intraoperative blood loss, and ICG-Krem were significantly associated with major complications. The median AUC of the five validation subsets was 0.878. The Hosmer-Lemeshow test confirmed no evidence of inadequate fit (*p* = 0.13, 0.19, 0.59, 0.59, and 0.73) on the fivefold cross-validation. The prediction model for major complications was as follows: −2.876 + 2.912 [LSM (>5.3 kPa)]+1.538 [ALBI score (>−2.28)]+0.531 [Intraoperative blood loss (>860 ml)]+0.257 [ICG-Krem (<0.10)].

**Conclusion:**

The proposed prediction model can be used to predict post-operative major complications in patients with HCC.

**Advances in knowledge:**

The proposed prediction model can be used in routine clinical practice to identify post-operative major complications in patients with HCC and to strategise appropriate treatments of HCC.

## Introduction

Liver resection is a first-line treatment for hepatocellular carcinoma (HCC),^1^ although the post-operative complications are serious problems. The previous studies report overall morbidity rate of open liver surgery range from 4.1 to 47.7%.^2,3^

The complications after liver resection are closely associated with background liver condition (such as cirrhosis, steatosis and active hepatitis) and limited residual liver volume.^4,5^ The indocyanine green clearance rate (ICG-K) of liver remnant (ICG-Krem) sensitively predicts the subclinical hepatic insufficiency.^6^ In addition, some biochemical markers, such as the Child‒Pugh score, platelet count, and the albumin‒bilirubin (ALBI) scores have been associated with post-operative complications.^7–9^

Magnetic resonance elastography (MRE) is among the most non-invasive and accurate tools for staging liver fibrosis,^10^ and previous studies have reported the usefulness of the liver-stiffness measurement (LSM) by MRE for predicting serious post-operative complications.^11–13^

To the best of our knowledge, however, no study has reported a model for predicting serious complications in patients with HCC. We hypothesised that the prediction model may improve diagnostic performance than the known methods, such as ICG-Krem, Child‒Pugh score, and ALBI score. This study aimed to create a novel prediction model, including MRE, to predict major complications after liver resection in patients with HCC.

## Methods and materials

### Patients’ characteristics and data collection

Our study was approved by the institutional review board of Nihon University School of Medicine Itabashi Hospital (Tokyo, Japan). This study was conducted in accordance with the principles of the Declaration of Helsinki. Each participant provided written informed consent for the study.

**Figure 1.**
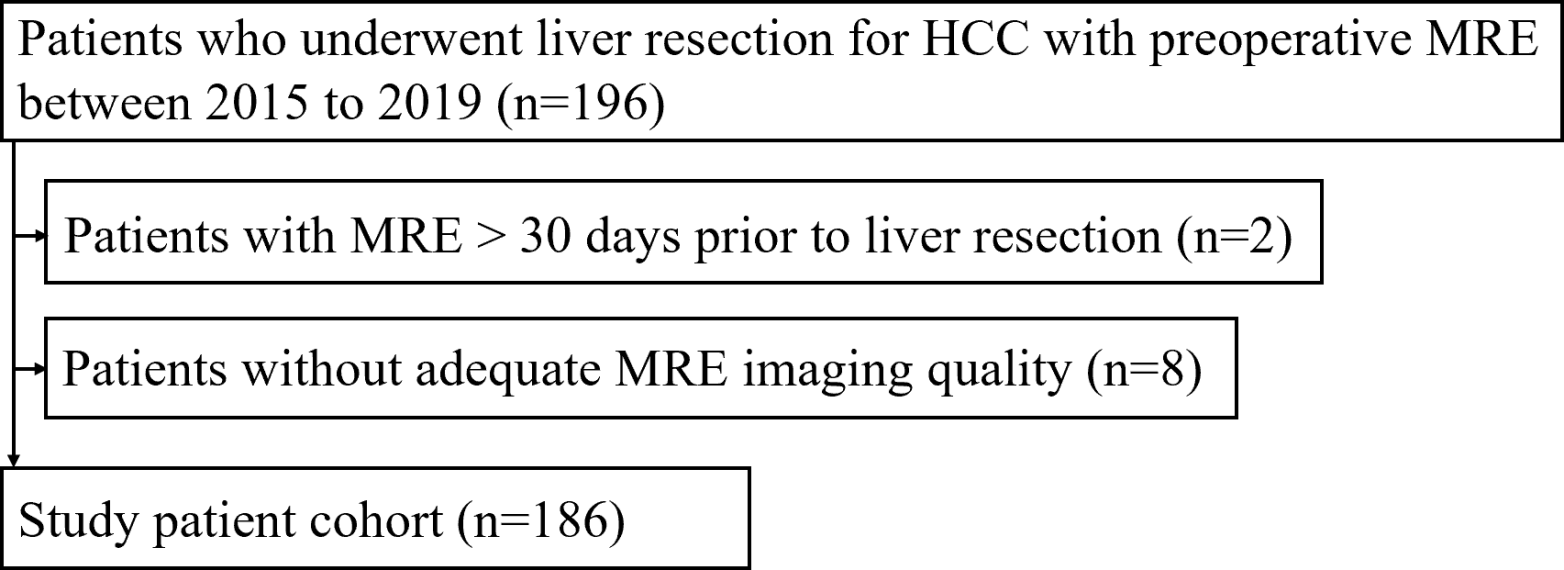
Flowchart of the study population. HCC, hepatocellular carcinoma; MRE, magnetic resonance elastography.

We included 196 consecutive patients, who underwent liver resection for HCC with pre-operative MRE between 2015 and 2019 (Figure 1). Patients who met the following criteria were excluded from our study: (1) patients who underwent MRE >30 days prior to liver resection; and (2) patient with inadequate MRE data because of the failure to generate satisfactory mechanical waves through the abdomen (Figure 1).

Patients’ characteristics (including history of liver resection, background liver disease, hepatic biochemical data, Indocyanine green clearance rate at 15 min (ICGR15), Child‒Pugh score), operative data (operative procedures, intraoperative blood loss, operation time, and complications), pathological data (pathological fibrosis stage, and neuroinflammatory activity grade) were collected. The ALBI score was calculated as follows: score = (log_10_ total-bilirubin [mg/dL]×17.1×0.66) + (albumin [g/dL] × 10 ×−0.085).^14^

Indications for liver resection and surgical procedures were based on Makuuchi’s criteria.^15^ Briefly, liver resection is contraindicated in patients with refractory hepatic encephalopathy and/or ascites. The extent of liver resection is prescribed by the serum total bilirubin level and ICGR15. Major liver resection was defined as resection of three or more Couinaud’s segments. Complications were categorised using Clavien‒Dindo classification,^16^ with major complications defined as Grade 3 or greater. Post-operative liver failure was defined as the presence of 50–50 criteria on post-operative day 5: INR >1.7 and total bilirubin >50 µmol l^−1^ (2.9 mg dl^−1^).^17^ Surgically resected specimens were histologically analysed. Pathological fibrosis stages (F0‒F4) and necroinflammatory activity grades (A0‒A3) were evaluated by two pathologists, based on the New Inuyama Classification.^18^

### Imaging techniques

MRE data were obtained using a 3.0-Tesla MR scanner (Discovery 750W; GE Medical Systems, Waukesha, WI). The parameters of the MRE were as described previously^13^ (Table 1). Magnetisation encoding gradient was 80 Hz. For MRE acquisition, 60 Hz mechanical shear waves with wave amplitude of 70% were applied to the liver with a proprietary passive driver placed over the right upper quadrant of the abdominal wall.^13^ After MRE scanning, the axial elastogram map and wave images were generated automatically on the operating console to evaluate quantitative liver stiffness in kilopascals (kPa), using commercially available software (MR Touch; GE Medical Systems). Iterative decomposition of water and fat with echo asymmetry and least squares estimation quantitation (IDEAL-IQ; Table 1)^19–21^ was obtained to estimate proton density fat fraction (PDFF) and the T2* component, using a modified Dixon method with advanced processing.

**Table 1.** Parameters of MRE and IDEAL IQ

	SE-EPI of MRE	Fast-GRE of IDEAL IQ
Strength of static magnetic field	3.0 Tesla	3.0 Tesla
TR/TE (msec)	800/58.9	7.7/1–5.1
Slice thickness (mm)	7	7
Flip angle (degrees)	90	4
Field of view (cm)	42	38
Matrix	64 × 64	160 × 160

Fast-GRE, Fast gradient echo sequence; IDEAL IQ, Iterative decomposition of water and fat with echo asymmetry and least squares estimation quantitation; MRE, MR elastography; SE-EPI, Spin-echo echo-planar imaging; TR/TE, Repetition time/echo time.

All of the pre-operative CT scan images were obtained with a reconstruction slice thickness of 1 mm following the multiphasic liver CT protocol, and the parameters of the CT were as described previously.^13^ For the portal dominant phases, 70 s fixed delays after the initiation of injection were adopted.

### Image analysis

Imaging measurements were performed by a sixth-year radiologist who was aware that the patients underwent liver resection, but was blinded to the clinical, surgical and pathological outcomes, and were confirmed by a senior radiologist.

**Figure 2.**
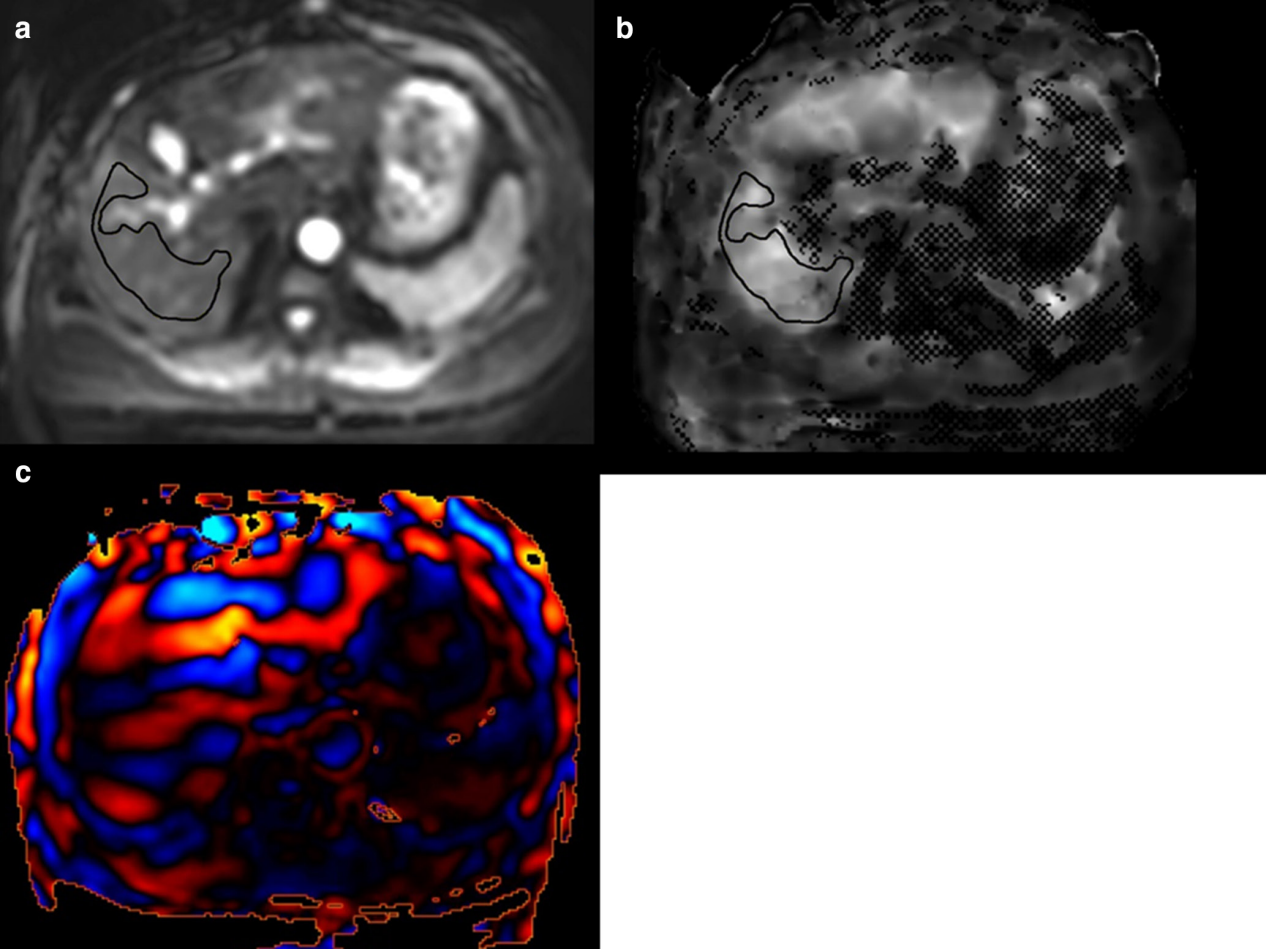
Measurements of liver stiffness measurement value by MR elastography. (a), Original echoplanar image of MR elastography (magnitude image). (b), A region of interest was placed on this elastogram. (c), Wave image.

Before the MRE quantitative analysis, the radiologist placed the regions of interest (ROIs) to avoid large hepatic vessels in the right lobe. This can be attributed to the vulnerability of the left lobe to cardiac motion artefacts.^22^ ROIs (largest possible) were manually placed on the elastogram, referring to the magnitude images and wave images. Moreover, areas involved in artefacts from motion or pulsation, areas with poor signal-to-noise ratios, regions below the driver, apparent tumours, (such as HCC, liver hemangioma and liver cyst), the liver edge, areas with inadequate wave propagation, and cross-hatching marks (unmeasurable area considering signal-to-noise ratios and wave parallelism) were avoided (Figure 2).^13,22–25^

**Figure 3.**
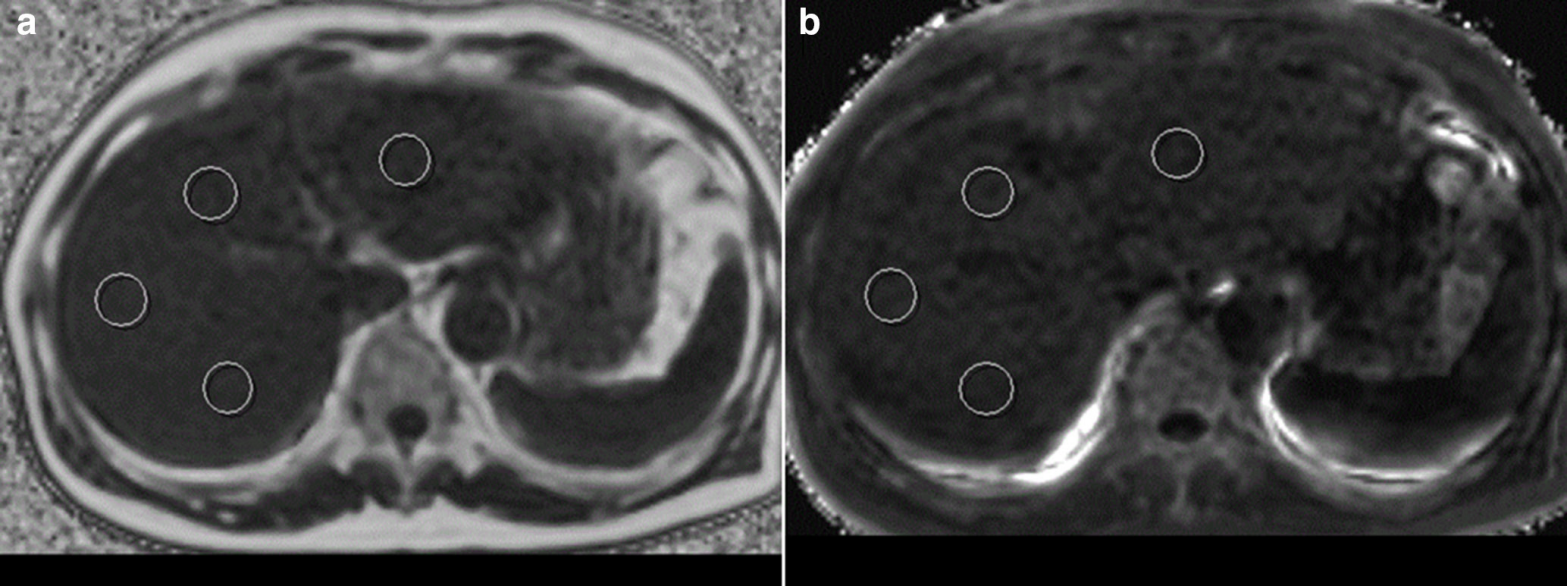
Measurements of PDFF and R2* value on fat fraction map and R2* map by iterative decomposition of water and fat with echo asymmetry and least squares estimation quantitation (IDEAL-IQ) (a), PDFF maps (magnitude image). (b), R2* maps. PDFF, proton density fat fraction.

Four ROIs (350–450 mm^2^) in the anterior, posterior, medial, and lateral liver segments were drawn at the hilar level on PDFF and on the R2* maps derived from the IDEAL IQ images (Figure 3).^26^ A commercially available picture archiving and communication system (SYNAPSE, Fujifilm Medical, Tokyo, Japan) was used to measure PDFF (%) and R2* (s^−1^). The averaged PDFF (%) and R2* (s^−1^) of the four ROIs were recorded.

The total liver volume was obtained by measuring the liver volume using CT volumetry (CTV), with a volume analyser system (Synapse Vincent, Fujifilm, Tokyo, Japan). CTV was reconstructed from 1 mm slice thickness CT images in the portal venous dominant phases.

### Calculating the indocyanine green clearance rate of liver remnants (ICG-Krem)

ICG was administered intravenously at a dose of 0.5 mg/kg as a routine liver function test, within 3 weeks before the surgery. ICG-K were measured by sampling at the following three time points: 5 min,10 min, and 15 min after injection. The method for calculating ICG-K has been described previously.^6^

Briefly, ICG-Krem consisted of the ICG-K, total liver volume (TLV) (mL), tumour volume (mL) and the weight of the resected specimen (g).^6^ The TLV was defined as the volume of the normal liver parenchyma after excluding tumorous tissues, and was calculated using the CTV. The tumour volume was based on the radius of the x, y and z axis, obtained from the cut surface of the specimen. The weight of the liver tissue was estimated at 1 g/1 ml of the parenchymal volume. The actual future liver remnant was eventually calculated using the following formula:

TLV (mL)−weight of the resected specimen (g) + tumour vol (mL)

### Statistical analysis

Continuous variables were expressed as medians with ranges and were compared between the major complications and non-major complications groups by using *t*-tests or Mann‒Whitney *U* tests, as appropriate. Categorical variables were compared between groups by using Fisher’s exact test. The correlation coefficient between LSM and fibrosis factors was calculated using Spearman’s correlation test. We analysed the receiver operating characteristic (ROC) curve for predicting major complications and calculated the area under the curve (AUC) among predictive factors in all patients.

For easier application to the prediction model, most continuous variables were converted to categories while performing the multivariable logistic regression analysis: advanced age (≥65 years), body mass index (>30 kg/m^2^), platelet count (<150×10^9^  l^−1^),^8^ PT-INR (>1.10), total bilirubin (>1.2 mg dl^−1^), AST (>39 U l^−1^), ALT (>45 U l^−1^), albumin (>3.8 mg l^−1^), ALBI score (>−2.28),^9^ hyaluronic acid (>200 ng ml^−1^),^27^ ICGR15 (>15%), ICG-Krem (<0.10),^6^ tumour diameter (>50 mm), intraoperative blood loss (>860 ml),^28^ LSM value (>5.3 kPa),^11^ PDFF value (>5%),^29^ R2* value (>60 s^−1^).

The prediction model for major complications was built and internally validated using fivefold cross-validation. While performing the fivefold cross-validation,^30^ we randomly divided all data into five equal-sized data sets. We intended to use four data sets for the development, remaining one data set for validation, over all possible permutations. For developing the prediction model, candidate predictors with *p* < 0.15 in univariate analyses among the four data sets were set for the stepwise multivariate logistic regression. To avoid multicollinearity, either was excluded from the input to the stepwise regression for a Kendall rank correlation coefficient >0.7. For validating the model, we analysed the ROC curve for the remaining data set, and calculated the AUC. The calibration was assessed by the Hosmer‒Lemeshow test, with *p* < 0.10 indicating an inadequate fit.^31^ Through the cross-validation process, we repeated the analysis five times (folds), with each of the five data sets used exactly once as the validation subset. The AUC was calculated for each of the five analyses, using only the respective data set. The AUCs statistics of the five validation subsets were subsequently aggregated into median, minimum, and maximum. The prediction model was constructed by averaging the significant regression coefficient values obtained from the five regression models.

The univariate and multivariate logistic regression were performed by SPSS Statistics v. 27.0 (IBM Corporation, Armonk, NY), and the ROC analysis of cross-validation were performed using R v. 4.0.2 statistical software. Two-sided *p* values < 0.05 were considered statistically significant.

## Results

### Patient characteristics

In total, 186 patients with HCC who underwent liver resection and pre-operative MRE were identified. Patient characteristics are summarised in Table 2. No patient was Child‒Pugh classification Grade C. ICGR15 and ALBI score was significantly larger in the major complications group than in the non-major complications group (*p* = 0.013 and *p* = 0.027, respectively). ICG-Krem and platelet count was significantly smaller in the major complications group than in the non-major complications group (*p* = 0.010 and *p* = 0.014, respectively). The median of the actual future liver remnant was 1163 ml (range: 687–2007 ml).

**Table 2.** Patient characteristics

	All patients (*n* = 186)
Age, years	68 (42–86)
Mele, n (%)	156 (83.9)
Female, n (%)	30 (16.1)
Body mass index, kg/m^2^	22.9 (15.5–37.3)
Background liver disease, n (%)	
Hepatitis B virus infection	58 (31.2)
Hepatitis C virus infection	72 (38.7)
The others	56 (30.1)
Haemoglobin, g/dL	13.8 (8.8–17.3)
Platelet count, 10^9^ l^−1^	161 (47–409)
PT-INR	1.00 (0.83–1.34)
Total bilirubin, mg/dL	0.65 (0.20–1.38)
AST, U/L	33 (12–137)
ALT, U/L	30 (6–150)
Albumin, g/L	4.2 (2.8–5.4)
Hyaluronic acid, ng/mL	83 (9–649)
ICGR15, %	13.3 (1.9–33.0)
ICG-Krem	0.127 (0.056–0.269)
ALBI score	−2.85 (-3.86–-1.50)
Child‒Pugh score, n (%)	
5 (class A)	171 (91.9)
6 (class A)	15 (8.1)
Type of liver resection, n (%)	
Limited resection	146 (78.5)
Segmentectomy	16 (8.6)
Sectionectomy	13 (7.0)
Major resection	11 (5.9)
Operative data	
Solitary tumour, n (%)	150 (80.6)
Tumour diameter, mm	28 (9–167)
Operation time, min	286 (107–714)
Transection time, min	58 (0–169)
Blood loss, mL	215 (14–2494)
Fibrosis stage	
F4	45 (24.2)
F3–4	85 (45.7)
Imaging data	
LSM value, kPa	4.21 (1.53–9.23)
PDFF value, %	2.82 (0.86–12.2)
R2* value, s^−1^	29.2 (9.8–59.5)
Major complications	43 (23.1)

ALBI, Albumin-bilirubin; ALT, Alanine aminotransferase; AST, Aspartate aminotransferase; ICG-Krem, The indocyanine green clearance rate of liver remnant; ICGR15, Indocyanine green retention rates at 15 min after injection; LSM, Liver stiffness measurement; PDFF, Proton density fat fraction; PT-INR, Prothrombin time-International normalised ratio.

Note: Continuous variables are expressed as median (range), if not specified. Categorical variables are expressed as number of patients.

### Operative data and post-operative complications

Operative data were shown in Table 2. Limited resection (non-anatomic resection), segmentectomy (Couinaud’s segment), sectionectomy, and major liver resection were performed in 146 (78.5%), 16 (8.6%), 13 (7.0%), and 11 (5.9%) of the 186 patients, respectively (Table 2).

Major complications occurred in 43 (23.1%) of the 186 patients (Table 3). Intraoperative blood loss was significantly larger in the major complications group than in the non-major complications group (*p* < 0.001). No patient had post-operative liver failure or mortality within 90 days.

**Table 3.** Post-operative major complications

Grade	n	Details	
Grade Ⅲa	41	Bile leakage	13
		Ascites	12
		Pleural effusion	7
		Wound infection	5
		Intra-abdominal infection	2
		Pneumothorax	1
		Angina	1
Grade Ⅲb	2	Post-operative bleeding	1
		Bile leakage	1
Grade Ⅳa	0		
Grade Ⅳb	0		
Grade Ⅴ	0		
Total	43		

Note: Post-operative complications are categorised according to the Clavien-Dindo classification.

Pathological fibrosis stages F0, F1, F2, F3, and F4 and necroinflammatory activity grades A0, A1, A2, and A3 of the background liver were observed in 9 (4.8%), 50 (26.9%), 42 (22.6%), 40 (21.5%), and 45 (24.2%) patients and in 9 (4.8%), 104 (55.9%), 71 (38.2%), and 2 (1.1%) patients, respectively.

### Quantitative MRE and IDEAL IQ data

The median LSM value was 4.21 kPa (range: 1.53–9.23 kPa) (Table 2). LSM in each fibrosis grade group was 2.10 kPa (range: 1.53–3.64 kPa), 3.29 kPa (range: 1.93–6.74 kPa), 3.86 kPa (range: 2.88–7.28 kPa), 4.16 kPa (range: 3.19–7.54 kPa), and 5.96 kPa (range: 3.49–9.23 kPa) for F0, F1, F2, F3, and F4 stages, respectively. The LSM was significantly higher in the major than in the non-major complications group in the development cohort (*p* < 0.001). The LSM correlated significantly with the liver fibrosis pathological stage (*r* = 0.614, *p* < 0.001). The median PDFF and R2* values were 2.82% and 29.2 s^−1^ (range: 0.86–12.2% and 9.8–59.5 s^−1^, respectively). These values did not differ significantly between the major and non-major complications groups (*p* = 0.19, *p* = 0.21, respectively) in the development cohort. No significant difference in LSM was seen between groups with necroinflammatory activity grades A2–3 and with A0–1 (*p* = 0.53), between groups with grades A1–3 or A0 (*p* = 0.34), between groups with PDFF ≥2.82% (median value) or <2.82% (*p* = 0.19), or between those with R2*≥29.2 s^−1^ (median value) or <29.2 s^−1^ (*p* = 0.41).

### Predictive factors for major complications in all 186 patients

**Figure 4.**
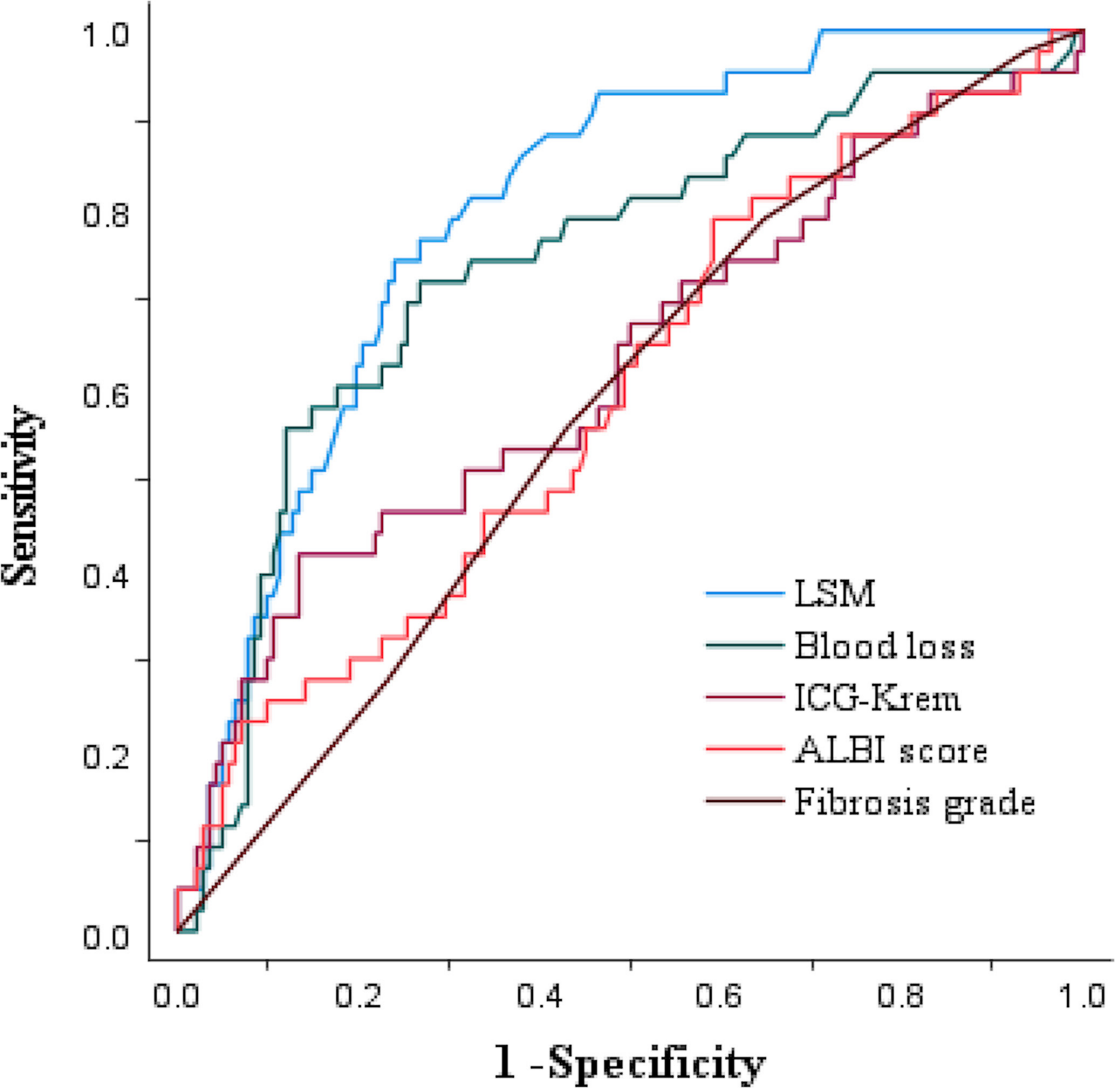
ROC curve for LSM value, Intraoperative blood loss, ICG-Krem, ALBI score, and fibrosis grade in all 186 patients. ALBI, albumin–bilirubin; ICG-Krem, indocyanine green clearance rate of liver remnant; LSM, liver stiffness measurement; ROC, receiver operating characteristic.

Based on the ROC curve analysis in 186 patients, the AUCs (95% confidence interval) of LSM, intraoperative blood loss, ICG-Krem, fibrosis grade, and ALBI score were 0.802 (0.734–0.870), 0.741 (0.653–0.830), 0.628 (0.526–0.729), 0.597 (0.501–0.694), and 0.580 (0.487–0.673), respectively (Figure 4).

### Cross-validation for development of a prediction model for major complications

Table 4 summarises the univariable analysis of predictive factors of major complications in each data set. The stepwise multivariate logistic regression analysis revealed LSM (>5.3 kPa), ALBI score (>−2.28), intraoperative blood loss (>860 ml) and ICG-Krem (<0.10) as independent factors for major complications (Table 5). Table 5 outlines the constant, explanatory variables, and regression coefficient values in each prediction models, built from the development data sets.

**Table 4.** Univariable analysis of predictive factors of major complications in each data set

	Data sets		Data sets		Data sets		Data sets		Data sets	
	2,3,4, and 5		1,3,4, and 5		1,2,4, and 5		1,2,3, and 5		1,2,3, and 4	
	OR	*p*	OR	*p*	OR	*p*	OR	*p*	OR	*p*
Age (≥65 years)	0.99	0.98	1.38	0.46	1.48	0.37	1.28	0.55	1.43	0.39
Mele	1.40	0.57	1.71	0.36	2.31	0.17	2.11	0.26	2.22	0.23
Body mass index (>30 kg/m2)	0.50	0.52	0.91	0.91	0.41	0.40	0.63	0.57	0.79	0.77
Hepatitis virus infection	0.52	0.14^a^	0.86	0.74	0.81	0.61	0.69	0.36	0.81	0.59
Platelet count (<150×10^9^ l^−1^)	2.37	0.042^*a*^	2.43	0.030^*a*^	1.76	0.16	1.60	0.22	1.78	0.13^*a*^
PT-INR (>1.10)	1.72	0.35	2.22	0.15	1.84	0.26	1.41	0.52	1.31	0.64
Total bilirubin (>1.2 mg dl^−1^)	1.70	0.54	1.05	0.96	0.67	0.72	0.47	0.49	1.12	0.89
AST (>39 U l^−1^)	3.40	0.004^*a*^	3.91	0.001^*a*^	2.60	0.018^*a*^	3.11	0.003^*a*^	3.46	0.001^*a*^
ALT (>45 U l^−1^)	2.12	0.082^*b*^	1.67	0.24	2.07	0.088^b^	1.37	0.45	1.60	0.25
Albumin (>3.8 g l^−1^)	2.00	0.12^*c*^	2.08	0.11^c^	1.30	0.58	1.37	0.47	1.87	0.17
ALBI score (>−2.28)	4.46	0.008^*a*^	7.47	0.001^*a*^	2.87	0.070^*a*^	2.91	0.055^*a*^	3.76	0.017^*a*^
Hyaluronic acid (>200 ng ml^−1^)	1.69	0.29	1.83	0.21	1.02	0.97	1.04	0.94	0.99	>0.99
ICGR15 (>15 %)	2.37	0.04^*a*^	2.25	0.046^*a*^	1.80	0.14^*a*^	1.50	0.29	2.5	0.017^*a*^
ICG-Krem (<0.10)	5.35	<0.001^*a*^	3.98	0.005^*a*^	4.52	0.002^*a*^	3.36	0.010^*a*^	3.96	0.003^*a*^
Child‒Pugh score 6	2.35	0.12^*c*^	3.57	0.016^*c*^	1.63	0.40	1.55	0.42	3.19	0.043^*c*^
Major resection	1.96	0.26	2.32	0.27	1.81	0.38	1.76	0.31	2.16	0.19
Multiple tumour	2.48	0.048^*a*^	2.30	0.078^*a*^	2.08	0.12^*a*^	2.65	0.019^*a*^	4.14	0.001^*a*^
Tumour diameter (>50 mm)	0.88	0.81	0.89	0.83	0.86	0.76	0.87	0.76	0.78	0.59
Intraoperative blood loss (>860 ml)	3.96	0.033^*a*^	1.95	0.30	1.14	0.88	1.75	0.40	3.06	0.092^*a*^
LSM value (>5.3 kPa)	27.8	<0.001^*a*^	11.0	<0.001^*a*^	11.4	<0.001^*a*^	13.6	<0.001^*a*^	13.8	<0.001^*a*^
PDFF value (>5 %)	0.95	0.92	1.52	0.33	1.50	0.36	1.21	0.65	1.26	0.57
R2* value (>60 sec^−1^)	0.78	0.63	1.17	0.74	1.17	0.74	0.75	0.53	0.74	0.87

ALBI, Albumin-bilirubin; ALT, Alanine aminotransferase; AST, Aspartate aminotransferase; ICG-Krem, The indocyanine green clearance rate of liver remnant; ICGR15, Indocyanine green retention rates at 15 min after injection; LSM, Liver stiffness measurement; OR, Odd ratio; PDFF, Proton density fat fraction; PT-INR, Prothrombin time-International normalised ratio.

a The candidate predictors were set for stepwise in multivariate logistic regression.

b The kendall rank correlation coefficient between the candidate predictors and AST was greater than 0.7.

c The kendall rank correlation coefficient between the candidate predictors and ALBI score was greater than 0.7.

**Table 5.** Prediction models of major complications from development data sets

	Constant	Regression coefficient values							
		LSM		ALBI score		Blood loss		ICG-Krem	
		>5.3 kPa	*p*	>−2.28	*p*	>860 ml	*p*	<0.10	*p*
Data sets 2,3,4, and 5	−4.249	4.086	<0.001	1.899	0.030	2.655	0.011		
Data sets 1,3,4, and 5	−2.668	2.483	<0.001	2.204	0.003				
Data sets 1,2,4, and 5	−2.459	2.350	<0.001					1.284	0.020
Data sets 1,2,3, and 5	−2.458	2.720	<0.001	1.484	0.030				
Data sets 1,2,3, and 4	−2.545	2.921	<0.001	2.105	0.002				

ALBI, albumin**-**bilirubin; ICG-Krem, The indocyanine green clearance rate of liver remnant; LSM, liver stiffness measurement.

**Figure 5.**
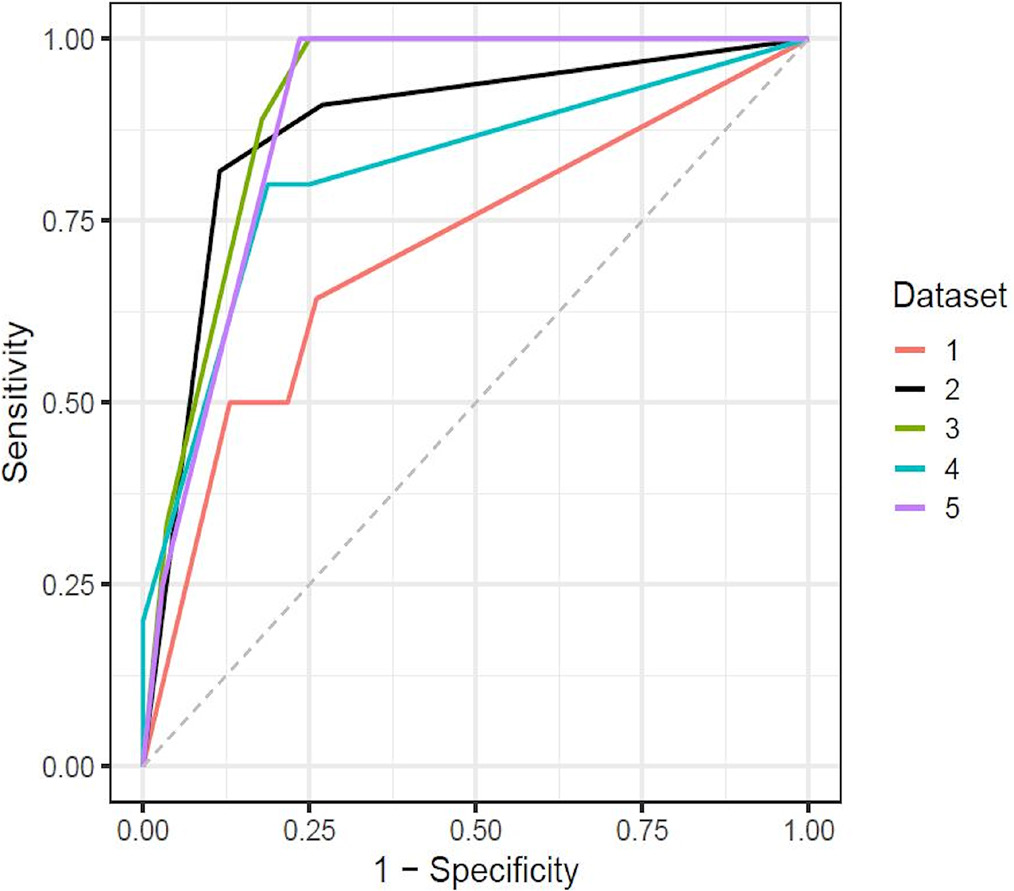
Receiver operating characteristic curves of the prediction models for major complications in fivefold cross-validation. Each line indicates the ROC curve of the validation data set. The median AUC of the five validation subsets was 0.878 (data set 2, black line). AUC, Area under the receiver operating characteristic curve; ROC, receiver operating characteristic.

Table 6 summarises the diagnostic performance of prediction models in five-fold cross-validation. Figure 5 depicts the ROC curves of the prediction models for major complications in fivefold cross-validation. The median AUC of the five validation subsets was 0.878 (minimum 0.708; maximum 0.911) (Table 6 and Figure 5). The Hosmer-Lemeshow test confirmed no evidence of inadequate fit (*p* = 0.13, 0.19, 0.59, 0.59, and 0.73) on the fivefold cross-validation (Table 6).

**Table 6.** Diagnostic performance of prediction models in fivefold cross-validation

Validation data set	Development data sets	AUC (95% CI)	Sensitivity (%)	Specificity (%)	*p^a^*
Data set 1	Data sets 2,3,4, and 5	0.708 (0.528–0.889)	64.3	73.9	0.73
Data set 2	Data sets 1,3,4, and 5	0.878 (0.746–1)	81.8	88.5	0.13
Data set 3	Data sets 1,2,4, and 5	0.911 (0.819–1)	100	75.0	0.19
Data set 4	Data sets 1,2,3, and 5	0.819 (0.589–1)	80.0	81.3	0.59
Data set 5	Data sets 1,2,3, and 4	0.897 (0.788–1)	100	76.5	0.59

AUC, Area under the curve; 95% CI, 95% confidence interval.

a *p* values were determined using the Hosmer-Lemeshow test.

### The prediction model for major complications

Based on the five prediction models from the development data sets (Table 5), the constant and the regression coefficient values of five regression models were averaged.

The prediction model for major complications was as follows:

Log (P/1-P)=−2.876+2.912 [LSM (>5.3 kPa)]+1.538[ALBI score (>−2.28)]+0.531[Intraoperative blood loss (>860 ml)]+0.257[ICG-Krem <0.10]. P was predicted as the probability of major complications following liver resection in patients with HCC.

## Discussion

In this study, we established the prediction model using LSM by MRE (>5.3 kPa), ALBI score (>−2.28), intraoperative blood loss (>860 ml), and ICG-Krem (<0.10) for estimating the risk of post-operative major complications in patients with HCC. Comparing each parameter (*e.g.* LSM, ICG-Krem) in all 186 patients, the mean AUC and 95% CI of LSM [0.802 (0.734–0.870)] was higher than conventional prediction factors including ICG-Krem [0.628 (0.526–0.729)] without overlapping in 95% CIs. The reproducibility of the proposed model was validated by cross-validation method because the median AUC of the five validation subsets (AUC: 0.878) was higher than AUCs of any parameter alone in all 186 patients. To our knowledge, this is the first report indicating that the prediction model may be useful for predicting major complications after liver resection in patients with HCC. Despite the need for an external validation, the prediction model can be used in routine clinical practice to identify high risk for post-operative complications in patients with HCC, and to select appropriate treatment strategies.

MRE has been accepted as one of the most non-invasive accurate tools for liver fibrosis staging.^10,32^ In this study, LSM correlated significantly with the pathological stage of liver fibrosis. Several studies have shown that LSM by MRE is an independent pre-operative risk factor for major complications after liver resection.^11–13^ The previous studies show that increased liver stiffness can make hepatic resection more difficult.^33,34^ The severity of liver fibrosis directly correlates with the amount of intraoperative blood loss.^11^ In our study, intraoperative blood loss was significantly larger in the major complications group than in the non-major complications group (*p* < 0.001). Our study results are in agreement with previous study that demonstrated the relationship between intraoperative blood loss and post-operative major complications.^28^ The higher rate of major complications in patients with higher LSM using MRE could also be explained by the presence of portal hypertension in some patients. Ronot et al^35^ demonstrated that LSM using MRE could predict the presence of severe portal hypertension. Bruix et al^36^ demonstrated a correlation between portal hypertension and post-operative complications following liver resection. Therefore, the degree of portal hypertension may explain our results.

LSM can potentially be affected by parenchymal inflammation, steatosis, and cholestasis. Hence, the aforementioned factors may confound liver fibrosis evaluation by LSM.^37–39^ Therefore, we examined these confounding factors. PDFF is extremely sensitive and specific for classifying the hepatic steatosis grade.^40,41^ However, we found no significant difference in LSM between groups with PDFF value ≥median value and <median value. Similarly, no significant difference in LSM was shown between patients with a necroinflammatory activity grade A2–3 and those with grade A0–1, and between those with grade A1–3 and those with grade A0. There were no patients with cholestasis in this study.

In the present study, ALBI grade was an independent risk factor for major complications, which was consistent with previous reports that indicated that the ALBI grade used for assessing liver function capacity could predict short-term outcomes after liver resection.^7,9^ ALBI grade is useful in clinical practice, because it can be calculated based on only blood test results. Thus, adding the ALBI grade to LSM by non-invasive MRE has clinical value.

Herein, the AUC of ICG-Krem for major complications in all 186 patients was 0.628, consistent with a previous study.^6^ While ICG-Krem was an independent risk factor for major complications, ICGR15 was not. This finding was consistent with previous reports that demonstrated ICG-Krem as a reliable predictor of the risk of post-operative subclinical hepatic insufficiency,^6^ and that ICGR15 was not a significant risk factor for short-term outcomes following liver resection.^42,43^ This finding can be explained by the fact that ICG-Krem not only consisted of ICG-K data but also that of the actual future liver remnant and total liver volume, and that the ICGR15 was not sensitive for the detection of early hepatic impairment.^44,45^

Our study had some limitations. First, selection bias could not be avoided because of the retrospective study design. Thus, a prospective study with a large number of patients is required to confirm our study results. Second, the number of patients in our study was relatively small and the prediction of post-operative major complications may not be clinically representative, because of single-centre cohorts. A multicentre study with an increased number of patients is desirable. Third, we did not evaluate the external validation of the prediction model, thus necessitating an external validation. Fourth, the prediction model requires LSM, and MRE needs additional equipment, such as a passive driver.

In conclusion, the prediction model, which included LSM by MRE, ALBI score, intraoperative blood loss, and ICG-Krem, can be useful in predicting the risk of post-operative complications before liver resection in patients with HCC. Despite the importance of an external validation, the prediction model may reduce the risk of surgery, and facilitate changes in the strategy for HCC treatment in patients at a higher risk of post-operative complications.
